# Chronic angiotensin receptor activation promotes hepatic triacylglycerol accumulation during an acute glucose challenge in obese-insulin-resistant OLETF rats

**DOI:** 10.1007/s12020-021-02834-7

**Published:** 2021-07-29

**Authors:** Jose A. Godoy-Lugo, Max A. Thorwald, David Y. Hui, Akira Nishiyama, Daisuke Nakano, Jose G. Soñanez-Organis, Rudy M. Ortiz

**Affiliations:** 1 School of Natural Sciences, University of California, Merced, CA USA; 2 Pathology and Laboratory Medicine, University of Cincinnati, Cincinnati, OH USA; 3 Department of Pharmacology, Kagawa University Medical School, Kagawa, Japan; 4 Universidad de Sonora, Departamento de Ciencias Químico Biológicas y Agropecuarias, Navojoa, Sonora Mexico; 5 Present Address: Leonard Davis School of Gerontology, University of Southern California, Los Angeles, CA USA

**Keywords:** Angiotensin receptor blocker, ARB, Insulin resistance, Liver steatosis, Metabolic syndrome, NAFLD

## Abstract

**Purpose:**

Angiotensin receptor blockers (ARBs) can ameliorate metabolic syndrome (MetS)-associated dyslipidemia, hepatic steatosis, and glucose intolerance, suggesting that angiotensin receptor (AT1) over-activation contributes to impaired lipid and glucose metabolism, which is characteristic of MetS. The aim of this study was to evaluate changes in the lipid profile and proteins of fatty acid uptake, triacylglycerol (TAG) synthesis, and β-oxidation to better understand the links between AT1 overactivation and non-alcoholic fatty liver disease (NAFLD) during MetS.

**Methods:**

Four groups of 25-week-old-rats were used: (1) untreated LETO, (2) untreated OLETF, (3) OLETF + angiotensin receptor blocker (ARB; 10 mg olmesartan/kg/d × 8 weeks) and (4) OLETF ± ARB (MINUS; 10 mg olmesartan/kg/d × 4 weeks, then removed until dissection). To investigate the dynamic shifts in metabolism, animals were dissected after an oral glucose challenge (fasting, 3 and 6 h post-glucose).

**Results:**

Compared to OLETF, plasma total cholesterol and TAG remained unchanged in ARB. However, liver TAG was 55% lesser in ARB than OLETF, and remained lower throughout the challenge. Basal CD36 and ApoB were 28% and 29% lesser, respectively, in ARB than OLETF. PRDX6 abundance in ARB was 45% lesser than OLETF, and it negatively correlated with liver TAG in ARB.

**Conclusions:**

Chronic blockade of AT1 protects the liver from TAG accumulation during glucose overload. This may be achieved by modulating NEFA uptake and increasing TAG export via ApoB. Our study highlights the contributions of AT1 signaling to impaired hepatic substrate metabolism and the detriments of a high-glucose load and its potential contribution to steatosis during MetS.

## Introduction

Non-alcoholic fatty liver disease (NAFLD) afflicts 25% of the world’s population and is projected to increase due to the increasing prevalence of diabetes and obesity [1, 2]. The metabolic syndrome (MetS) comprises several disorders that raise the risk of NAFLD [3]. MetS is identified when at least three of the following criteria are present simultaneously: (a) abdominal obesity, (b) elevated circulating triacylglycerol (TAG), (c) reduced HDL cholesterol levels, (d) hypertension, and (e) increased fasting glucose ± insulin resistance [4]. Obesity and insulin resistance are factors that contribute to the development of MetS [3].

Inappropriately elevated angiotensin II (Ang II) and over-activation of its target receptor, AT1, contribute to impaired hepatic lipid metabolism [5], development of fatty liver [6], and the development of insulin resistance [7–9]. Thus, elevated Ang II is regarded as a risk factor for NAFLD [10]. Chronic infusion of Ang II increased circulating insulin, TAG, NEFA, and liver TAG synthesis in rats [11, 12]. Ang II-mediated activation of AT1 may overload the liver by increasing plasma NEFA, decreasing fatty acid oxidation, and promoting de novo lipogenesis [5]. Angiotensin receptor blockers (ARB) displace Ang II from its receptor [13], inhibiting Ang II signaling. While ARBs are widely used to ameliorate MetS-related hypertension [14], they also improve markers of inflammation [15, 16], components of the lipid profile, and hepatic lipid accumulation in animal studies and clinical trials [17–21]. However, the mechanisms contributing to these improvements are not fully understood, especially in relationship to AT1 signaling.

The sequestration of hepatic NEFA is mainly dependent on membrane fatty acid transporters, which include the fatty acid transport protein (FATP) family [22], cluster of differentiation 36 (CD36), and caveolins [23, 24]. In the liver, FATP5 and FATP2 are the most abundantly expressed of the 6 members of the FATP family. Knockdown [25] and knockout (KO) [26] of FATPs ameliorated hepatic steatosis. These studies also demonstrated a positive correlation between FATP levels and NEFA uptake in the liver [25, 26]. Hepatic expression of CD36 is typically low but increases with fatty liver [27, 28]. A positive correlation between plasma insulin levels and hepatic CD36 expression was observed in insulin-resistant patients with steatosis [29].

Recent studies suggest that peroxiredoxin 6 (PRDX6) may provide an anti-steatotic effect during fatty liver disease, mainly through elimination of oxidants [30]. PRDX6 is a special member of its family because it is bifunctional, having both peroxidase and phospholipase A2 activity [31]. PRDX6 protected the liver against damage and mitochondrial dysfunction induced by oxidative stress during ischemia-reperfusion [32]. NAFLD is associated with excessive oxidant production, which is linked with impaired mitochondrial function [33]. Yet, studies addressing hepatic PRDX6 changes in a MetS model do not exist.

Hepatic lipid accumulation is the hallmark of NAFLD [3, 34], which may result from an imbalance between lipid acquisition and their disposal or oxidation [27]. Nonetheless, the mechanisms that promote the development of steatosis, and the potential AT1 signaling involved, are not completely understood [27, 35]. In the present study we investigated liver lipid substrates and proteins for NEFA uptake, TAG and VLDL cholesterol synthesis, and fatty acid oxidation in the liver of rats treated with ARB and their changes in response to an acute glucose challenge, as well as the potential effects of the removal of ARB treatment.

## Methods

All experimental procedures were reviewed and approved by the institutional animal care and use committees of the Kagawa Medical University (Japan) and the University of California, Merced (USA). Phenotypical data (i.e., body mass, oral glucose tolerance test curves, insulin levels, and blood pressure) has been previously published [36] with that study focused on redox signaling in the heart in response to a glucose challenge. The current study is unique and complements the previous one by extending the examination of the dynamic changes in hepatic lipid metabolism in response to a glucose challenge, which represents a nutrient overload that may appear in Western diets [37, 38]. We have included previously published [36] body mass and plasma angiotensin II levels (Table 1) to better illustrate our model and the efficacy of the ARB treatment. Additionally, we examine the potential detriments of non-compliance with ARB treatment after the initial treatment has stopped (legacy effect) [39]. Low compliance has proven to be a challenge when treating patients with hypertension, dyslipidemia, and diabetes [40, 41] and assessing ARB-legacy effects may provide a better understanding of the detriments to removal of treatment [42, 43].

**Table 1:** Mean (±SD) end of study body mass and plasma angiotensin II

	LETO	OLETF	ARB	MINUS
Body Mass (g)	465 ± 29	610 ± 31^a^	598 ± 36^a^	602 ± 28^a^
Plasma AngII (fmol/ml)	58.9 ± 3.1	68.6 ± 11.6	211.3 ± 27.9^a,b^	65.2 ± 10.2^c^

^a^Significant difference from LETO (*P* < 0.05)

^b^Significant difference from OLETF (*P* < 0.05)

^c^Significant difference from ARB (*P* < 0.05)

In this study, we approach the obtained results from two perspectives: (1) the static outcomes following the chronic treatment with ARB and its removal (potential legacy effect) and (2) the dynamic response to a glucose challenge following chronic treatment with ARB and its removal (non-compliance). In all cases, comparisons are made to a lean, strain-control.

### Animals

OLETF rats are reported to develop insulin resistance and hyperglycemia by 17 weeks of age [44, 45]. For that reason, male, age matched, 17-week-old, lean strain-control Long Evans Tokushima Otsuka (LETO; 428 ± 8 g) and obese Otsuka Long Evans Tokushima Fatty (OLETF; 536 ± 6 g) rats (Japan SLC Inc., Hamamatsu, Japan) were used. Rats were assigned to the following groups (*n* = 5–7 animals/group/time point): (1) untreated LETO, (2) untreated OLETF, (3) OLETF + angiotensin receptor blocker (*ARB*; 10 mg olmesartan/kg/d × 8 weeks) [46], and (4) OLETF ± ARB (*MINUS*; 10 mg olmesartan/kg/d × 4 weeks, then removed the last 4 wks prior to dissection). ARB (Daiichi-Sankyo, Tokyo, Japan) was administered by oral gavage suspended in carboxymethyl cellulose (CMC) to conscious rats and untreated rats were gavaged with CMC only. Animals were maintained in groups of two to three animals per cage, given access to water and standard laboratory chow (MF; Oriental Yeast Corp., Tokyo, Japan), and maintained under controlled temperatures (23–24 °C) and humidity (~55%) with a light-dark cycle of 12–12 h.

For dissections, the 25-week-old rats were fasted for 12 h ± 15 min. To investigate the dynamic response to a glucose challenge, animals were dissected at baseline (T0, fasting) and after 3 (T3) and 6 h (T6) after a glucose load by gavage (2 g glucose/kg mass). Comparisons of the T0 (fasting baseline) data from each group characterized the static changes in response to chronic ARB treatment and its removal. The glucose challenge was performed to evaluate the acute, dynamic changes in metabolism and cellular responses. Initiation of the overnight fasts and the glucose gavages were staggered to meet the exact dissections timepoints. Animals were decapitated and trunk blood collected in vials containing EDTA (Sigma-Aldrich, EDS) and proteinase inhibitor cocktail (Sigma-Aldrich, P2714). Livers were snap frozen in liquid nitrogen and kept at −80 °C until analyzed.

### Biochemical analyses & markers of hepatic NAFLD

Insulin, glucose, plasma total cholesterol (TC), TAG, and NEFA concentrations as well as collagen type IV (COL-4) were measured using the commercially available reagents: Insulin Rat ELISA Kit (Thermo Fisher Scientific, ERINS), Autokit glucose (Fujifilm Wako Diagnostics, 997-03001), Total Cholesterol E (Fujifilm Wako Diagnostics, 999-02601), L-Type Triglyceride M (Fujifilm Wako Diagnostics, 994-02891 and 990-02991), HR Series NEFA-HR (2) (Fujifilm Wako Diagnostics, 999-34691, 995-34791, 991-34891, and 993-35191), and CIV ELISA (MyBioSource, MBS732756), respectively, following manufacturer’s instructions. All samples were analyzed in duplicate and only accepted values that fell within a percent coefficient of variability of less than 10% for all measurements.

### Western blot analyses

A 25 mg piece of frozen liver was homogenized in phosphate buffer for a two-step extraction of cytoplasm and plasma membrane proteins. Briefly, phosphate buffer (50 mM potassium phosphate) (Fisher Scientific, P290 and P285) was used to homogenize the liver, then centrifuged at 15,000 × *g* to recover the supernatant containing the cytoplasmic fraction. Then, 50 mM potassium phosphate buffer +1% Triton X-100 (Millipore-Sigma, T8787) was used to homogenize the pellet, which contained the plasma membrane fraction. The pellet homogenate was centrifuged at 15,000 × *g*, and the plasma membrane contents recovered from the supernatant. The buffers contained 3% protease inhibitor cocktail (Sigma-Aldrich, P2714) to help prevent protein degradation. The protein content of the fractions was quantified using the Bradford assay (Bio-Rad Laboratories, 5000203). Total protein (5–10 μg) was resolved in 10% Tris-HCL SDS gels. Proteins were electroblotted onto 0.45-μm polyvinyl difluoride (PVDF) (Millipore-Sigma, IPVH00010) membranes by semi-wet transfer using the Mini Gel Tank and Blot Module Set (Invitrogen, NW2000). Intercept blocking buffer (Li-Cor, 927-60001, 927-70001) was used to block the membranes then incubated 16 h with the corresponding primary antibody (diluted 1:1,000-1:2,000) against glycerol-3-phosphate acyltransferase 1 (GPAM) (abcam, ab69990), FATP5 (Invitrogen, PA5-42028), diacylglycerol O-acyltransferase 1 (DGAT1) (Thermo Fisher, PA5-79150), ApoB (Apolipoprotein B) (Thermo Fisher, PA5-86950), CD36 (Thermo Fisher, PA1-16813), FATP2 (Thermo Fisher, PA5-30420), carnitine palmitoyl transferase I (CPT1A) (Proteintech, 15184-1-AP), Peroxisomal acyl-coenzyme A oxidase 1 (Acox1) (Thermo Fisher, PA5-76341), PRDX6 (Proteintech, 13585-1-AP), and β actin (Cell Signaling Technology, 3700S) (diluted 1:5,000). Membranes were washed, incubated with IRDye 800CW anti-rabbit (Li-Cor, 926-32213) and/or 680RD donkey anti-mouse IgG secondary antibodies (Li-Cor, 926-68072) (diluted 1:20,000), and rewashed. Blots were visualized using the Odyssey system (Li-Cor) and quantified using the Image Studio Lite ver. 5.2 (Li-Cor) using β actin as a loading control. Plasma membrane and cytosolic fractions were tested for purity against Na^+^/K^+^ ATPase antibody (Abcam, ab76020) (diluted 1:40,000) and alpha tubulin (Abcam, ab52866) (diluted 1:40,000), respectively.

### Statistics

Data was tested for normality using the Shapiro–Wilk test [47]. Means ± standard deviation (SD) were compared by two-way ANOVAs when analyzing datasets with all timepoints (T0, T3, T6) and groups, and one-way-ANOVA when analyzing datasets with only basal levels (T0 only). Means were considered significantly different at *p* < 0.05 using Tukey’s HSD. Correlations were calculated using the Pearson r coefficient [48, 49], using the means and SD obtained from each group and timepoint, computed using the displayed individual values in each figure. Area under the curve (AUC) analyses were calculated using the area under the concentration curve in batch designs [50]. Outliers were calculated and removed using the ROUT test [51] and one outlier was replaced with the mean of the corresponding group [52]. All statistical analyses were performed with GraphPad Prism 8.4.3 software (GraphPad Prism, La Joya, CA).

## Results

To better recognize the difference between static (i.e., chronic ARB treatment reflected by differences at T0) and dynamic (i.e., during the acute glucose load) responses, the results were separated into two sections contained within each subheading, designated respectively to the response.

### ARB normalized the secretion of insulin in response to glucose

Previous studies have shown that ARBs decrease plasma glucose [53, 54] and report improvements in insulin resistance [55], yet other studies report no changes [56, 57]. Therefore, to better address the existing discrepancies in the literature, we measured plasma glucose and insulin statically over time and dynamically in response to the glucose load.

#### Static Changes

**Fig. 1:**
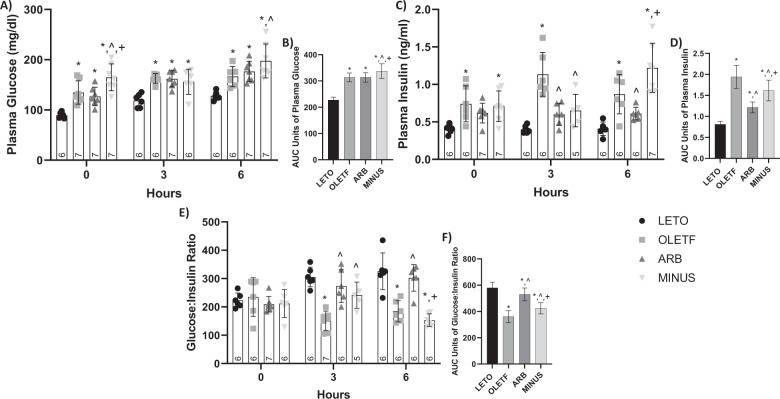
ARB decreased plasma insulin after a glucose load. Mean ± SD values of plasma glucose (**A**), AUC of plasma glucose (**B**), plasma insulin (**C**), AUC of plasma insulin (**D**), glucose:insulin ratio (**E**), and AUC of glucose:insulin ratio (**F**), during the glucose challenge in Long Evans Tokushima Otsuka (LETO), Otsuka Long Evans Tokushima Fatty (OLETF), OLETF + ARB (ARB; ARB x 8 weeks), and OLETF ± ARB (MINUS, ARB x 4 weeks, then removed x 4 weeks) rats. Number at bottom of the bar indicates n per group. *Significant difference from LETO (P *<* *0.05). ^ Significant difference from OLETF (P* *<* *0.05). + Significant difference from ARB (P* *<* *0.05)*

Basal circulating glucose levels were 34%, 30%, and 46% greater in OLETF, ARB, and MINUS, respectively, than LETO (Fig. 1A). While levels in MINUS were 22% and 29% greater than OLETF and ARB, respectively (Fig. 1A). Basal plasma insulin was 80% and 73% greater in OLETF and MINUS, respectively, compared to LETO (Fig. 1C). The basal glucose-to-insulin ratios were not different among the groups (Fig. 1E).

#### Dynamic changes

During the acute glucose challenge, circulating glucose levels were 40% and 30% greater in OLETF, 36% and 37% greater in ARB, and 31% and 55% greater in MINUS, when compared to LETO at T3 and T6, respectively (Fig. 1A). Levels in MINUS at T6 were 18% and 12% greater than OLETF and ARB, respectively (Fig. 1A). Mean glucose AUC was 38%, 38%, and 48% greater in OLETF, ARB and MINUS, respectively, when compared to LETO, whereas mean AUC in MINUS was 8% greater than OLETF and ARB (Fig. 1B).

At T3, plasma insulin levels were 183% greater in OLETF compared to LETO, and 89% and 74% lesser in ARB and MINUS, respectively, compared to OLETF (Fig. 1C). At T6, levels were 75% and 145% greater in OLETF and MINUS, respectively, when compared to LETO (Fig. 1C). Plasma insulin levels were 30% and 50% lesser in ARB compared to OLETF and MINUS, respectively (Fig. 1C). Mean insulin AUC was 140%, 52%, and 99% greater in OLETF, ARB, and MINUS, respectively, when compared to LETO (Fig. 1D). Mean AUCs were 37% and 17% lesser in ARB and MINUS, respectively, compared to OLETF, whereas MINUS AUC was 31% greater compared to ARB (Fig. 1D).

At T3, the glucose-to-insulin ratio was 50% lesser in OLETF compared to LETO, while ratios in ARB and MINUS were 45% and 37% greater than OLETF (Fig. 1E). At T6, ratios in OLETF and MINUS were 43% and 53% lesser than LETO, while the ARB ratio was 39% greater than OLETF. The ratio on MINUS remained 49% lesser than ARB (Fig. 1E). Mean glucose-to-insulin ratio AUC was 38%, 9%, and 15% lesser in OLETF, ARB, and LETO, respectively, compared to LETO (Fig. 1F). Mean ratios in ARB and MINUS were 32% and 15% greater, respectively, compared to OLETF, while ratio on MINUS remained 25% lesser compared to ARB (Fig. 1F).

Contrasting with other studies [53, 54], ARB did not change the glucose levels, yet it lowered insulin levels during the glucose challenge and ameliorated the glucose-to-insulin ratio as seen previously [55, 56].

### ARB increased plasma NEFAs without changing TAG levels

COL-4 has been shown to accurately detect NAFLD with levels representing the degree of severity [58, 59].

#### Static Changes

**Fig. 2:**
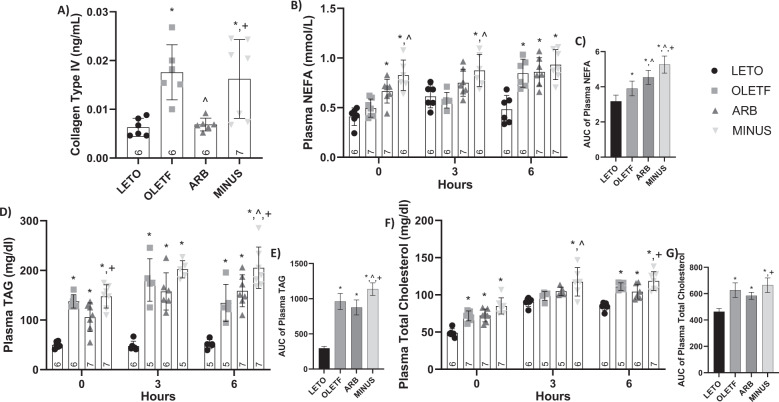
ARB increased plasma NEFAs without changing TAG levels. Mean ± SD values of COL-4 (**A**) plasma NEFA (**B**), AUC of plasma NEFA (**C**), plasma triacylglycerol (**D**), AUC of plasma triacylglycerol (**E**), plasma total (**F**) and AUC (**G**) of total cholesterol, during the glucose challenge in Long Evans Tokushima Otsuka (LETO), Otsuka Long Evans Tokushima Fatty (OLETF), OLETF + ARB (ARB; ARB x 8 weeks), and OLETF ± ARB (MINUS, ARB x 4 weeks, then removed x 4 weeks) rats. Number at bottom of the bar number indicates *n* per group. *Significant difference from LETO (P *<* *0.05). ^ Significant difference from OLETF (P* *<* *0.05). + Significant difference from ARB (P* *<* *0.05)*

COL-4 levels were 178% and 157% greater in OLETF and MINUS, respectively, compared to LETO (Fig. 2A). Levels were 155% lesser in ARB compared to OLETF (Fig. 2A). Levels in MINUS were intermediate between ARB and OLETF with levels 135% greater than ARB (Fig. 2A). These results suggest that liver fibrosis is decreased with ARB, while the removal of the treatment led to an intermediary phenotype associated with loss of most of the ARB benefits.

While it is known that ARB treatment improves circulating NEFA, TAG, and TC [12, 17, 60], these variables were measured here to better interpret the changes as they relate to the changes in the proteins associated with their metabolism and to ascertain the effects of the glucose challenge on lipid metabolism. *Static Changes*: Basal plasma NEFA was 38% and 50% greater in ARB and MINUS, respectively, compared to LETO (Fig. 2B). Plasma TAG was 174%, 110%, and 194% greater in OLETF, ARB and MINUS, respectively, compared to LETO, while MINUS remained 40% greater than ARB (Fig. 2D). Plasma TC was 47%, 47%, and 73% greater in OLETF, ARB, and MINUS, respectively, compared to LETO (Fig. 2F).

#### Dynamic changes

During the glucose challenge, plasma NEFA in MINUS was 43% and 30% greater than LETO and OLETF at T3, respectively (Fig. 2B). At T6, levels in OLETF, ARB and MINUS were 76%, 79%, and 93% greater, respectively, compared to LETO (Fig. 2B). Mean plasma NEFA AUC was 17%, 42%, and 64% greater in OLETF, ARB and MINUS, respectively, compared to LETO (Fig. 2C). Mean AUC in ARB and MINUS was 17% and 29%, respectively, greater than OLETF, while MINUS remained 14% greater than ARB (Fig. 2C).

At T3, plasma TAG levels in OLETF, ARB, and MINUS remained 275%, 227%, and 320% greater, respectively, than LETO (Fig. 2D). At T6, levels in OLETF, ARB, and MINUS were 162%, 211%, and 302% greater, respectively, than LETO. While MINUS remained 53% and 29% greater compared to the OLETF and ARB groups, respectively (Fig. 2D). Mean plasma TAG AUC was 222%, 195%, and 285% greater in OLETF, ARB, and MINUS, respectively, compared to LETO, while MINUS remained 20% and 30% greater than ARB and OLETF, respectively (Fig. 2E).

At T3, plasma TC levels were 23% and 98% greater in MINUS than LETO and OLETF, respectively (Fig. 2F). At T6, levels were 30%, 22%, and 38% greater in OLETF, ARB, and MINUS, respectively, compared to LETO, while MINUS remained 13% greater than ARB (Fig. 2F). Mean plasma TC AUC was 21%, 22%, and 39% greater in OLETF, ARB, and MINUS, respectively, compared to LETO, and MINUS remained 15% and 14% greater than OLETF and ARB, respectively (Fig. 2G).

ARB plasma NEFA AUC was greater than OLETF, while plasma TAG AUC remained unchanged. On the other hand, MINUS AUCs were all greater than ARB, which demonstrates the detrimental effect of treatment removal. AT1 activation triggers mechanisms that induce insulin resistance and hypertension [42], the progression insulin resistance in these animals is exponential during the its youth [44]. In our study, we treated our rats during its early stages of MetS (17 weeks of age) and the more developed stage (21 weeks of age), when the rat presents pronounced hepatic steatosis [44, 61]. During the initial phase of AT1 blockade (4 weeks of treatment), the measured parameters may be ameliorated. Though when the treatment is removed, the impact of Ang II signaling may worsen, acting stronger, which may be explained as a drug-derived re-bound effect [62].

### ARB protected against the glucose-induced accumulation of hepatic TAG

We measured part of the hepatic lipid profile to confirm this effect and further investigate it during a glucose challenge.

#### Static Changes

**Fig. 3:**
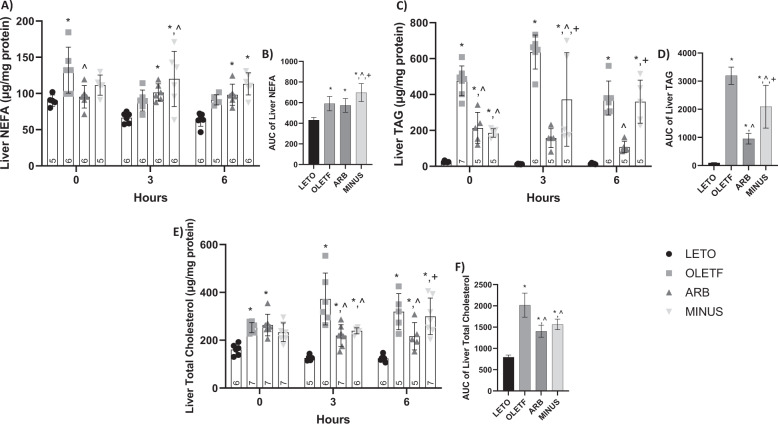
ARB conferred protection against the glucose-induced increase of hepatic TAG. Mean ± SD values of liver NEFA (**A**), AUC of NEFA (**B**), triacylglycerol (**C**), AUC of triacylglycerol (**D**), total cholesterol (**E**), and AUC of total cholesterol (**F**), during the glucose challenge in Long Evans Tokushima Otsuka (LETO), Otsuka Long Evans Tokushima Fatty (OLETF), ARB (OLETF + angiotensin receptor blocker x 8 weeks), and MINUS (OLETF + angiotensin receptor blocker x 4 weeks, then removed x 4 weeks) rats. Number at bottom of the bar number indicates *n* per group. *Significant difference from LETO (P *<* *0.05). ^ Significant difference from OLETF (P* *<* *0.05).* ^*+*^*Significant difference from ARB (P* *<* *0.05)*

Basal liver NEFA was 47% greater in OLETF than LETO, while ARB was 39% lesser than OLETF (Fig. 3A). Liver TAG was 1880%, 787%, and 670% greater in OLETF, ARB, and MINUS, respectively, compared to LETO, while levels in ARB and MINUS remained 55% and 61% lesser than OLETF (Fig. 3C). Liver TC was 58% and 64% greater in OLETF and ARB, respectively, compared to LETO (Fig. 3E).

#### Dynamic Changes

During the glucose challenge, liver NEFA levels in ARB and MINUS were 53% and 81% greater than LETO at T3, respectively, while MINUS remained 33% greater than OLETF (Fig. 3A). Mean liver NEFA AUC was 40%, 38%, and 62% greater in OLETF, ARB, and MINUS, respectively, compared to LETO, while MINUS remained 15% and 17% greater than OLETF and ARB, respectively (Fig. 3B).

At T3, liver TAG levels were 4790% and 2760% greater in OLETF and MINUS, respectively, compared to LETO, and remained 41% lesser in MINUS compared to OLETF, and 138% greater than ARB (Fig. 3C). At T6, levels were 2600% and 1040% greater in OLETF and MINUS, respectively, compared to LETO, while ARB levels were 72% lesser than OLETF, and MINUS levels were 70% greater than ARB (Fig. 3C). Mean liver TAG AUC was 3260%, 900%, and 1935% greater in OLETF, ARB, and MINUS, respectively, compared to LETO. ARB and MINUS mean liver TAG remained 40% and 70% lesser, respectively, than OLETF, while MINUS remained 50% greater than ARB (Fig. 3D).

At T3, liver TC levels were 195%, 74%, and 90% greater in OLETF, ARB and MINUS, respectively, compared to LETO, while ARB and MINUS remained 41% and 36% lesser than OLETF, respectively (Fig. 3E). At T6, levels were 159%, 76%, and 143% greater in OLETF, ARB, and MINUS, respectively, compared to LETO. ARB remained 32% lesser than OLETF and MINUS remained 28% greater than ARB (Fig. 3E). Mean liver TC AUC was 145%, 71%, and 89% greater in OLETF, ARB, and MINUS, respectively, compared to LETO, while ARB and MINUS remained 30% and 23% lesser than OLETF, respectively (Fig. 3F).

ARB improved hepatic NEFA and TAG levels and protected it from TC accumulation during the glucose challenge. Notably, the MINUS group showed ameliorated basal hepatic TAG accumulation; however, during the glucose challenge, TAG and NEFA increased, along with their respective AUC. We attributed this to the re-bound effect [62], as thoroughly discussed above, here MINUS demonstrates more detrimental effects following non-compliance.

### ARB decreased basal the abundance of CD36, and β-oxidation proteins in the liver following the glucose challenge

Because the liver-specific deletion of AT1 reduces hepatic steatosis [63], we wanted to assess the potential for decreased sequestration of NEFA as a contributing factor. Therefore, we measured the protein abundance of CD36, FATP5, and FATP2, which are the main liver NEFA transporters [22, 64, 65].

#### Static Changes

**Fig. 4:**
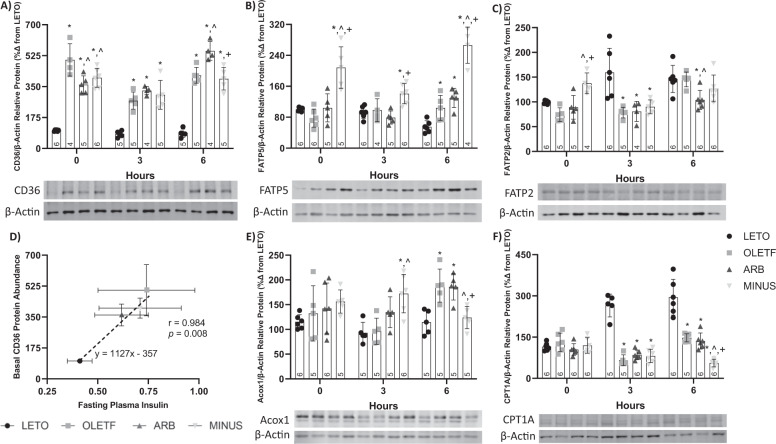
ARB decreased basal membrane CD36 abundance in the liver. Western blot results, mean ± SD values for relative liver membrane protein abundance of CD36 (**A**), FATP5 (**B**), FATP2 (**C**), results for Pearson’s correlation test of CD36 vs Plasma Insulin (**D**), Acox1 (**E**), and CPT1A (**F**), during the glucose challenge in Long Evans Tokushima Otsuka (LETO), Otsuka Long Evans Tokushima Fatty (OLETF), OLETF + ARB (ARB; ARB x 8 weeks), and OLETF ± ARB (MINUS, ARB x 4 weeks, then removed x 4 weeks) rats. Number at bottom of the bar number indicates *n* per group. *Significant difference from LETO (P *<* *0.05). ^ Significant difference from OLETF (P* *<* *0.05).* ^*+*^*Significant difference from ARB (P* *<* *0.05)*

Basal CD36 protein abundance was 79%, 53%, and 74% greater in OLETF, ARB and MINUS, respectively, compared to LETO and remained 28% and 20% lower in ARB and MINUS, respectively, compared to OLETF (Fig. 4A). CD36 abundance and basal levels of fasting plasma insulin were significantly and positively correlated (*r* = 0.9836, *p* = 0.008) (Fig. 4D). FATP5 protein abundance was 52%, 62%, and 50% greater in MINUS than LETO, OLETF, and ARB, respectively (Fig. 4B). FATP2 protein abundance was 46% and 35% greater in MINUS compared to OLETF and ARB, respectively (Fig. 4C).

#### Dynamic changes

During the glucose challenge, CD36 protein abundance was 70%, 75%, and 73% greater in OLETF, ARB, and MINUS, respectively, compared to LETO at T3 (Fig. 4A). At T6, protein abundance was 80%, 75%, and 73% greater in OLETF, ARB, and MINUS, respectively, compared to LETO (Fig. 4A). While ARB was 25% greater than OLETF and 28% lesser than MINUS (Fig. 4A).

At T3, FATP5 protein abundance was 34% and 44% greater in MINUS than LETO and ARB, respectively (Fig. 4B). At T6, protein abundance was 47%, 57%, and 79% greater in OLETF, ARB and MINUS than LETO, and remained 60% and 51% greater in MINUS than OLETF and ARB, respectively (Fig. 4B).

At T3, FATP2 protein abundance was 51%, 50%, and 43% lesser in OLETF, ARB, and MINUS, respectively, compared to LETO (Fig. 4C). At T6, protein abundance was 29% and 28% lesser in ARB than OLETF and LETO, respectively (Fig. 4C).

Collectively, the ARB-induced decrease in basal CD36 may partially explain the amelioration in hepatic lipid accumulation [29] while the greater abundance in FATP2 and FATP5 in the MINUS group may be responsible of the increased TAG accumulation [66].

#### Static changes

On the other hand, Ang II signaling can increase hepatic lipids by decreasing fatty acid oxidation [5] and both Acox1 and CPT1 are essential mediators of lipid β-oxidation [23, 67]. We hypothesized that chronic blockade of AT1 increases β-oxidation. No basal changes in Acox1 (Fig. 4E) or CPT1A (Fig. 4F) were detected among the groups suggesting that AT1 activation at this stage of MetS is not sufficient to profoundly impair oxidation.

#### Dynamic changes

During the glucose challenge, Acox1 protein abundance was 46% and 44% greater in MINUS compared to LETO and OLETF, respectively (Fig. 4D). At T6, protein abundance was 39% and 38% greater in OLETF and ARB than LETO, respectively, and remained 34% and 33% lesser in MINUS than OLETF and ARB, respectively (Fig. 4D).

At T3, CPT1A protein abundance was 75%, 68%, and 81% greater in OLETF, ARB, and MINUS than LETO, respectively (Fig. 4F). At T6, protein abundance was 50%, 52%, and 81% lesser in OLETF, ARB, and MINUS than LETO, respectively, and remained 62% and 59% lesser in MINUS than ARB and MINUS, respectively (Fig. 4F). CPT1A abundance in MINUS tended to decrease (*r* = 0.9944, *p* = 0.07) in response to glucose over time.

The protein abundance for Acox1 and CPT1A in MINUS remained lower than OLETF and ARB at 6 h following the glucose challenge suggesting that the TAG accumulation in this group was partially attributed to a reduction in lipid oxidation. This distinct behavior further demonstrates the detriment of non-compliance that is masked by analyzing static changes alone.

### ARB decreased ApoB abundance but not proteins of TAG synthesis

Insulin can activate the liver-X-receptor, which promotes the transcription of genes of hepatic lipogenesis [68]. Previously, we demonstrated that ARB treatment decreased plasma insulin [69]. Therefore, we hypothesized that ARB treatment decreases TAG accumulation through suppression of TAG synthesis. The first committed step in TAG synthesis is catalyzed by glycerol-3-phosphate acyltransferase [70] while diglyceride acyltransferase (DGAT) mediates the last step of TAG synthesis [71].

#### Static Changes

**Fig. 5:**
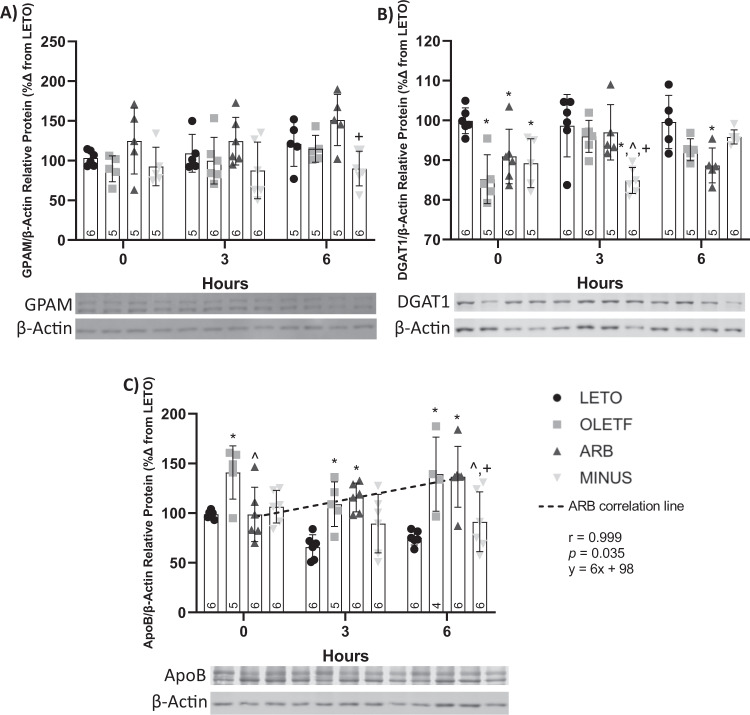
ARB decreased basal ApoB but not TAG synthesis protein levels in the liver. Western blot results, mean ± SD values for relative protein abundance of GPAM (**A**), DGAT1 (**B**), and ApoB (**C**), including Pearson’s correlation test of ApoB vs time, during the glucose challenge in Long Evans Tokushima Otsuka (LETO), Otsuka Long Evans Tokushima Fatty (OLETF), OLETF + ARB (ARB; ARB x 8 weeks), and OLETF ± ARB (MINUS, ARB x 4 weeks, then removed x 4 weeks) rats. Number at bottom of the bar number indicates *n* per group. *Significant difference from LETO (P *<* *0.05). ^ Significant difference from OLETF (P* *<* *0.05). + Significant difference from ARB (P* *<* *0.05)*

In our study, basal GPAM protein abundance remained unchanged (Fig. 5A), and DGAT1 abundance was 15%, 10%, and 11% lesser in OLETF, ARB, and MINUS, respectively, compared to LETO (Fig. 5B).

#### Dynamic changes

During the glucose challenge, no profound changes in GPAM were detected among groups or over time, except for a 40% decrease in MINUS at T6 when compared to ARB (Fig. 5A). In OLETF, GPAM abundance tended (*r* = 0.9940, *p* = 0.07) to increase in response to glucose over time. At T3, DGAT1 protein abundance was 14%, 11%, and 12% lesser in MINUS compared to LETO, OLETF and ARB, respectively, and at T6, was 11% lesser in ARB than LETO (Fig. 5B).

#### Static changes

TAGs can be used to form VLDL with ApoB, which is secreted from the liver [72] and hepatic secretion of VLDL increases with insulin resistance [73]. Therefore, we measured ApoB to gain insight into hepatic VLDL formation and the effects of ARB on its production.

#### Static Changes

Basal ApoB was 29% greater in OLETF compared to LETO, and 29% lesser in ARB than OLETF (Fig. 5C).

#### Dynamic changes

During the glucose challenge, ApoB protein abundance was 39% and 43% greater in OLETF and ARB, respectively, than LETO, at T3 (Fig. 5C). At T6, ApoB protein abundance remained 46% and 45% greater in OLETF and ARB than LETO, respectively, and was 34% and 33% lesser in MINUS compared to OLETF and ARB, respectively (Fig. 5C). ApoB levels increased linearly (*r* = 0.9985, *p* = 0.04) over 6 h in ARB (Fig. 5C).

Hepatic sequestration of NEFA and chylomicron remnants is routed for oxidation or TAG esterification. TAG is also produced from excess glucose through *de novo* lipogenesis [5]. In our study, the abundance of TAG synthesis proteins remained unchanged with ARB treatment, while its removal caused their abundance to decrease during the glucose challenge. Elevated Ang II can alter VLDL secretion [5] and here we demonstrated that ARB treatment decreased ApoB protein abundance, a key protein for VLDL assembly [72] suggesting that less VLDL is present or being made in the liver. Furthermore, the linear increase in ApoB during the glucose challenge suggests that TAG is incorporated into VLDL to be secreted from the liver, preventing TAG accumulation.

### Glucose stimulates PRDX6 abundance, which was inversely correlated to hepatic TAG in ARB

PRDX6 is a bifunctional protein with peroxidase and phospholipase A2 activity [31], which is associated with ameliorating NAFLD via its antioxidant capacity and PLA2-like activity [30, 74]. Because Ang II infusion may induce NAFLD through oxidant imbalance [6], we hypothesized that ARB treatment increases PRDX6 levels to potentially reduce oxidant imbalance.

#### Static changes

**Fig. 6:**
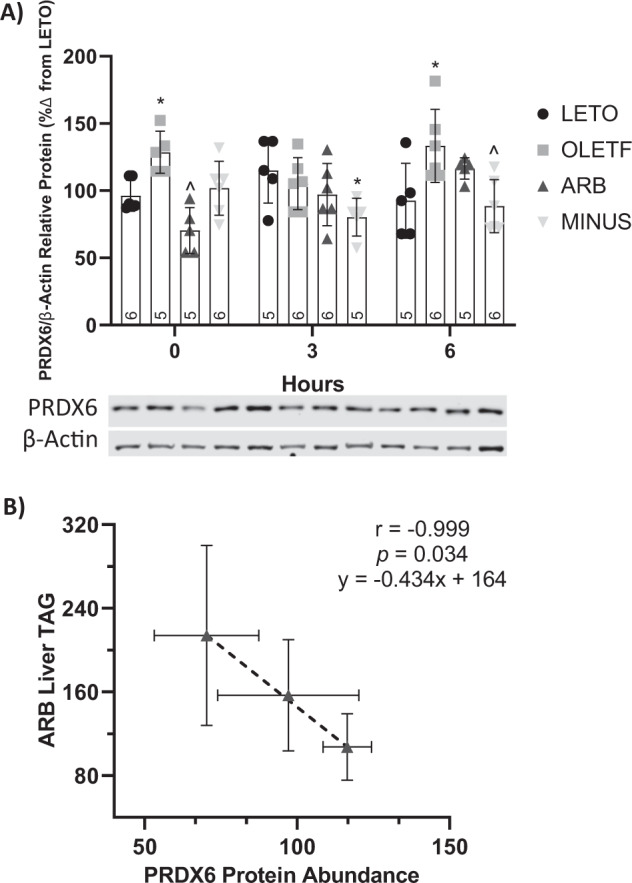
Hepatic PRDX6 protein decreased with ARB, and PRDX6 abundance correlated to intrahepatic TAG. Western blot results, mean ± SD values for relative protein abundance of PRDX6 (**A**) and results for Pearson’s correlation test of PRDX6 vs Liver TAG (**B**), during the glucose challenge in Long Evans Tokushima Otsuka (LETO), Otsuka Long Evans Tokushima Fatty (OLETF), OLETF + ARB (ARB; ARB x 8 weeks), and OLETF ± ARB (MINUS, ARB x 4 weeks, then removed x 4 weeks) rats. Number at bottom of the bar number indicates *n* per group. *Significant difference from LETO (P *<* *0.05). ^Significant difference from OLETF (P* *<* *0.05).* ^*+*^*Significant difference from ARB (P* *<* *0.05)*

Basal PRDX6 protein abundance was 25% greater in OLETF compared to LETO, and protein abundance was 45% lesser in ARB compared to OLETF (Fig. 6A).

#### Dynamic changes

During the glucose challenge, PRDX6 abundance in MINUS was 30% lesser than LETO, at T3 (Fig. 6A). At T6, protein abundance was 31% greater in OLETF compared to LETO, and 34% lesser in MINUS compared to OLETF (Fig. 6A). In ARB, PRDX6 abundance was significantly and negatively correlated with liver TAG levels (*r* = −0.9986, *p* = 0.04) (Fig. 6B), while PRDX6 protein abundance tended (*r* = 0.9956, *p* = 0.06) to positively correlate over time in response to the glucose challenge.

Notably, PRDX6 abundance was lower in the ARB group. Though the concept is new, increased PRDX6 expression protected mice from developing hepatic steatosis [30]. Accordingly, we found an inverse relationship between PRDX6 abundance and hepatic TAG levels, suggesting that PRDX6 abundance may present with decreased liver TAG levels and reduced hepatic steatosis. Additionally, the lower basal PRDX6 levels in ARB may reflect the lack of a need to stimulate antioxidant mechanisms, supported by improvements on redox proteins after ARB treatment [20, 36, 69], which may be reduced as the need is ameliorated.

## Discussion

NAFLD afflicts 25% of the world’s population [1]. Hepatic TAG accumulation is the hallmark of NAFLD [3, 34], with lipid accumulation in the liver resulting from an imbalance between lipid sequestration and its disposal or metabolism [27]. Nonetheless, the mechanisms promoting steatosis in MetS [27, 35, 75] in relation to Ang II signaling [5] are not completely understood. In our study, we investigated the effects of chronic AT1 blockade statically and the dynamic responses to a glucose challenge on hepatic lipid accumulation and proteins mediating NEFA uptake, TAG and VLDL cholesterol synthesis, and fatty acid oxidation in rats with MetS. Additionally, we investigated the legacy-effect of ARB treatment following its removal. We found a marked decrease on liver TAG that may be achieved by modulating NEFA uptake, through CD36, and increased TAG export via ApoB. The inverse relationship between PRDX6 abundance and hepatic TAG and the increasing levels of PRDX6 in response to the glucose challenge when hepatic TAG levels are decreasing suggests that PRDX6 may protect the liver from steatosis derived from TAG accumulation. Furthermore, our results are unique as they highlight the detrimental effects of treatment non-compliance through many contrasts between the ARB and MINUS groups.

OLETF rats are characterized by elevated Ang II before the onset of insulin resistance [76], suggesting that elevated Ang II may contribute to the development of insulin resistance [7]. Liver-specific deletion of the Ang II receptor (AT1) reduces hepatic steatosis [63], evidencing the contribution of AT1 signaling on hepatic lipid accumulation. TAG accumulation is a hallmark of NAFLD [3] and NEFA can be a precursor for TAG formation [5], while VLDL cholesterol can be assembled into larger molecules that may not leave the hepatocyte [72]. In the liver, ARB decreased basal NEFA and TAG indicative of an improvement in liver lipid metabolism in MetS. Conversely, hepatic TAG, NEFA, and TC were increased in the MINUS group during the glucose challenge, suggesting that non-compliance is associated with hepatic lipid accumulation in response to a glucose load, consistent with a Western diet. Thus, while most of the ARB-mediated benefits remained statically, they were lost during the glucose challenge.

Hepatic expression of CD36 is typically low although it increases with fatty liver disease [27, 28]. In our study, hepatic CD36 levels in LETO were remarkably lower than all OLETF groups and remained relatively low throughout the glucose challenge. ARB treatment decreased CD36 abundance and even after its removal the levels remained lesser than OLETF. Furthermore, fasting plasma insulin and basal CD36 membrane protein abundance were strongly correlated, consistent with the data in patients with steatosis [29] suggesting that the hyperinsulinemia associated with MetS contributes to hepatic lipid accumulation and dysregulation of lipid metabolism via up-regulation of hepatic CD36.

CD36 protein abundance was increased in the myocardium of hyperglycemic mice [77] and vascular lesions of hyperglycemic patients [78], suggesting that elevated glucose may also up-regulate the abundance of CD36. In the present study, liver CD36 was greater in ARB, ending the glucose challenge. The lack of a detectable changes CD36 in response to the glucose challenge in LETO suggests that, during healthy conditions, CD36 protein levels are maintained despite the hyperglycemia. Additionally, CD36 levels decrease initially at 3 h post-glucose in OLETF, ARB and MINUS, increasing further only in ARB at 6 h post-glucose, when elevated glucose levels have cleared circulation, suggesting that hepatic CD36 may not be stimulated by hyperglycemia during MetS. Thus, the changes in CD36 abundance in response to hyperglycemic conditions may be tissue-specific.

The FATP family facilitates the transport of NEFA into the cell. FATP5 and FATP2 are the most abundant in the liver [22]. Knockdown and KO studies of hepatic FATP proteins are correlated with NEFA uptake [25, 26, 79]. ARB treatment did not change the basal membrane abundance of either protein, but FATP2 abundance decreased at 6 h post-glucose in ARB, while CD36 was elevated in the same group, suggesting that increased CD36 may compensate for the decrease in FAT proteins in the liver. Non-compliance (MINUS) increased the FATPs expression, suggesting that the potential to sequester NEFA is increased, supported by its greater liver NEFA AUC.

Ang II-mediated signaling can increase hepatic lipid-overload by decreasing hepatic fatty acid oxidation [5]. Acox1 is a rate-limiting enzyme in peroxisomal lipid β-oxidation [67]. Acox1-deficient rodents exhibit sudden steatosis [80]. On the other hand, CPT1A converts fatty acyl-CoA to fatty acyl-carnitine for subsequent β-oxidation [81]. Human CPT1 expression is reduced during NAFLD [82], suggesting that decreased β-oxidation may be an important factor in the development of steatosis. In our study, ARB treatment did not change Acox1 or CPT1A protein abundance; however, during the glucose challenge, Acox1 abundance was greater in MINUS than OLETF and ARB, while at the end of the challenge, Acox1 abundance was lesser than OLETF and ARB. These changes suggest that the removal of ARB may have changed the sensitivity of Acox1 regulation to glucose. On the other hand, glucose induced an increase in CPT1A in the healthy LETO, which remained elevated after 6 h, suggesting that under normal conditions a healthy liver may increase lipid oxidation to help reduce TAG and NEFA accumulation in response to a glucose load. CPT1A levels in the OLETF, ARB and MINUS groups were substantially lower than LETO, however, they increased in the OLETF and ARB groups at 6 h post-glucose, while MINUS levels remained suppressed. In MINUS, the decreasing trend (*p* < 0.07) in CPT1A, but not Acox1, abundance suggests that an acute glucose load may reduce the hepatic β-oxidation capabilities and that CPT1A abundance may be more sensitive to a glucose load, following non-compliance.

Collectively, these data suggest that: (1) under normal, healthy conditions the potential to increase β-oxidation may be primarily via CPT1-mediated mechanisms, (2) MetS may be associated with impaired CPT1A-mediated β-oxidation, which is supported by the lack of CPT1A abundance increase during the glucose challenge in the OLETF, ARB and MINUS groups, and (3) that non-compliance (MINUS) may be associated with impaired β-oxidation via CPT1A to a greater extent than untreated (OLETF) conditions.

During insulin resistance, hyperinsulinemia may increase lipogenesis and TAG accumulation [83, 84]. Insulin can increase activation of the liver-X-receptor to promote hepatic lipogenesis [68]. GPAM [70] and DGAT [71] participate in the synthesis of TAG. Hepatic GPAM expression was elevated in patients with steatosis or NASH [85]. In our study, plasma insulin AUC was lower with ARB treatment, which corresponded with lower liver TAG AUC, yet GPAM abundance remained unchanged. This may be due to GPAM activity being regulated, more so than its abundance. On the other hand, DGAT deficiency in primary hepatocytes protected against increased lipid deposition by decreasing TAG synthesis [86]. Also, inhibition of DGAT1 protected against fatty liver in mice on a high-fat diet [87]. Yet during lipodystrophy, increased de novo hepatic fatty acid synthesis caused steatosis, independent of changes in DGAT1 [88], suggesting that static changes in DGAT1 may not have a significant contribution to TAG synthesis. The increasing trend (*p* < 0.07) in GPAM abundance in response to glucose in OLETF suggests that high glucose-loads may worsen hepatic lipid accumulation by stimulating TAG synthesis. Alternatively, the measures of GPAM and DGAT protein abundance may not be enough to accurately reflect changes in the activity of these enzymes [88, 89]. The maintenance of these relatively higher levels of hepatic TAG in the OLETF and MINUS groups may be a consequence of chronically elevated TAG synthesis, which may not be stimulated by the glucose load used in this study.

Alternatively, hepatic lipids can be mobilized through the secretion of VLDLs [72], which are a primary vehicle to transport synthesized TAG to circulation for utilization in peripheral tissues. Each VLDL particle contains one molecule of ApoB [73], suggesting that ApoB abundance is a reliable surrogate measure for relative changes of VLDL in the liver. Secretion of VLDL-TAG is increased in patients with NAFLD [90]. Also, it is suggested that VLDL particles from individuals with NAFLD may be larger and contain more TAG [72, 91], preventing them from leaving the cell. Therefore, the differences in particle size cannot be excluded as a contributing factor to the observed accumulation of hepatic TAG. In our study, ApoB abundance increased in OLETF and was decreased in ARB. This suggests that the elevated levels in OLETF may be necessary to bind the greater amount of free TAG, increasing the potential for shuttling it out of the cell. Alternatively, the increased levels of ApoB in OLETF may reflect accumulation due to large particles of VLDL, which cannot be shuttled out of the liver [72].

In response to the glucose challenge, ApoB abundance increased over time in ARB. This may be due to the increased synthesis of VLDL to further decrease TAG accumulation in the liver of the ARB group. This is further supported by increased plasma TAG, which translates to elevated circulating VLDL, since VLDL accounts for ~20% of the measured plasma TAG [92, 93]. Conversely, the low abundance of ApoB in MINUS at 6 h post-glucose suggests that the potential to shuttle TAG out of the cell in response to glucose may be impaired, and that the increased plasma TAG may come mainly from diet and increased lipolysis, contributing to greater levels of hepatic TAG, reflecting the detrimental effect of treatment removal. Ultimately, these data provide an additional mechanism by which hepatic TAG is regulated in response to nutrient loads.

Induction of CD36 with palmitic acid induced hepatocyte activation dependent on oxidative stress pathways, while CD36 KO reduced these adverse effects [24], suggesting that changes in CD36-mediated NEFA transport are associated with changes in redox balance in the liver. However, these relationships are not well-defined during MetS. PRDX6 is member of the PRDX family of proteins, which may protect against obesity-related pathologies, mainly through elimination of oxidants [94–96]. The C57BL/6J‐Tg transgenic mice with increased PRDX6 expression prevented the liver from developing steatosis [30], suggesting that PRDX6 may contribute directly to the regulation of hepatic lipid accumulation. PRDX6 KO mice on a high-fat diet increased levels of circulating alanine aminotransferase (ALT), a marker of hepatic injury that is associated with development of NASH [74]. In our study, basal PRDX6 abundance was greater in OLETF, while ARB treatment reduced this increase, suggesting that ARB treatment improved the redox status in the liver, and that increased levels of hepatic PRDX6 may reflect the need for improved redox balance in the liver. Glucose stimulated a trend (*p* < 0.06) for linear increase in the ARB group, suggesting that a glucose load may stimulate redox gene transcription factors to initiate antioxidant mechanisms in response to increased glucose levels. PRDX6 and liver TAG levels were negatively correlated in the ARB group, suggesting that either: (a) increasing hepatic TAG accumulation may decrease PRDX6 levels or (b) increasing PRDX6 reduces hepatic TAG accumulation. Yet, very limited information is available regarding the relationship between PRDX6 and liver TAG levels. Therefore, in the current study, deciphering which is the independent and dependent variable is not possible, though the novel finding is that there is a negative relationship between these two variables that has not been previously reported in a model of MetS. Although the negative correlation between PRDX6 and liver TAG can be thought as isolated, it may be meaningful for future research linking PRDX6 and NAFLD, where literature on this topic is scarce. Although the link between NAFLD and PRDX6 remains unclear [30, 74], our data supports the idea that PRDX6 may participate in protecting the liver from TAG accumulation.

In summary, these results demonstrate that chronic blockade of AT1, through ARB treatment, protects the liver from TAG accumulation, especially during a glucose load. This may be achieved by decreasing NEFA uptake and increasing TAG export via CD36 and FATP2, and ApoB, respectively. Additionally, treatment non-compliance reverted many of the potential benefits observed in ARB, which may leave the liver more susceptible to further lipid accumulation and injury over time. Finally, frequent acute glucose loads may contribute to increased hepatic lipid accumulation through the maintenance of TAG synthesis and impaired β-oxidation and cellular lipid export, to ultimately develop NAFLD in MetS.

## Data Availability

Associated data are available upon reasonable request.
